# Bread without Fermentation

**Published:** 1846-08

**Authors:** W. B. Herapath


*Bread without Fermentation.* By W. B. Herapath.

Having seen in the last week's *Lancet* an advertisement relating to the newly-invented process for the manufacture of bread without the aid of yeast or any other fermenting substance, perhaps I might me permitted to call the attention of the medical profession (and especially of that portion practising in our colonies) to this subject, by making a few remarks upon the process, and the numerous advantages it offers over that generally adopted.

From the influence which temperature has upon fermentation, great difficulty is constantly found in obtaining good yeast in tropical climates; and in long maritime voyages, other circumstances conspire to render this obstacle so insurmountable, that, after having fruitlessly tried numerous expedients, the attempt to obtain fresh bread has been almost given up in despair. The natives of our Indian colonies have either been driven to eat bread sour from the moment when it has been drawn from the oven, or to choose an equally disagreeable alternative, to digest dry, heavy and unwholesome biscuits. Fresh bread is a luxury equally unknown to the mariner and the colonist; consequently, the method now before us will prove an invaluable boon to both parties, as it will enable them to procure 'the staff of life' almost at a moment's warning.

Before entering upon the discussion of the improved process, perhaps it will be advisable to make a few remarks upon the theory of the ordinary method of 'rising the dough.' Your readers are of course aware that the essential ingredients for the manufacture of bread have hitherto been flour, water, and some substance capable of inducing fermentation. Yeast is commonly used, which consists of an immense quantity of peculiarly organized vegetable cells, having extraordinary powers of reproduction when placed in circumstances favourable to their development. Saccharine solutions, kept at a proper temperature, permit these cells to germinate rapidly, and during their growth, the sugar is converted into alcohol and carbonic acid gas, the latter of which is evolved in large quantities. Flour contains a certain proportion of sugar, which, therefore, in the manufacture of dough, becomes converted into spirit of wine, and the carbonic acid gas, during its escape in bubbles, inflates and lightens the dough, by separating its particles farther from each other; and then during baking, the dough is dried sufficiently for it to retain this porous form, after all gas has escaped.

Any other substance capable of eliminating gas would be equally efficacious in 'rising the dough,' as the porosity of bread simply depends upon the mechanical effect of the bubbles of gas upon the dough previously to baking.

The method adopted by the patentee of the 'prepared flour,' therefore, has this object in view—viz., to evolve by chemical decomposition, during the wetting of the flour, a sufficient quantity of carbonic acid gas to render the dough spongy, light, and elastic, so that the bread might be made porous, palatable, and digestible. He has perfectly succeeded in his object, and at the same time done so without communicating to the bread any flavour likely to excite the criticism of the most gastronomic individual; the articles introduced are so small in quantity, and so perfectly harmless in their physiological properties, that no one can ever object to make use of an article thus prepared. Some time ago he kindly permitted me to inspect his apparatus, and the whole process of preparing the flour, making the dough, and baking the bread upon this principle, and I can safely say, that I have never witnessed any operation in domestic economy so beautifully simple and efficacious. A few minutes suffice to mix the necessary ingredients with the flour, and then, simply by stirring up a little water with this mixture, and kneading the mass for a short time, it becomes dough, as spongy and elastic as if twelve hours had been consumed in its manufacture by the old method; in another hour it is drawn from the oven as a loaf equal to anything ever produced by the finest and sweetest yeast. It is not necessary that the ingredients should be mixed with the flour immediately before the bread is to be made; flour thus prepared might be kept in store for months, or years even, without undergoing more change than any other meal would under similar circumstances. I have eaten a loaf made from flour thus prepared eight months ago, and can testify to its sweetness and perfect flavour. Bread thus made has been kept for months without showing any sign of mouldiness or decay; it hardens and becomes dry, but continues sweet to the last.

It will be at once seen, from the simplicity of the process, that we are now perfectly independent of fermentation, and consequently of all the disadvantages attendant upon changes in temperature; for whether we desire to obtain bread in the depth of winter, or during the greatest heats of summer, in the polar regions, or in the torrid zone, our food will be as readily made, and as light and spongy, as if produced under the most advantageous circumstances, according to the ordinary process.—*Ibid.*

